# Mind-wandering in daily life in depressed individuals: An experience sampling study

**DOI:** 10.1016/j.jad.2024.08.111

**Published:** 2024-08-23

**Authors:** Matthew S. Welhaf, Jutta Mata, Susanne M. Jaeggi, Martin Buschkuehl, John Jonides, Ian H. Gotlib, Renee J. Thompson

**Affiliations:** a Department of Psychological & Brain Sciences, Washington University in St. Louis, USA; b School of Social Sciences, Health Psychology, University of Mannheim, Germany; c Center for Cognitive and Brain Health, Northeastern University, Boston, USA; d MIND Research Institute, USA; e University of Michigan, USA; f Department of Psychology, Stanford University, USA

**Keywords:** Mind-wandering, Major Depressive Disorder, Ecological momentary assessment, Time-lagged analyses

## Abstract

**Background:**

A diagnostic criterion for Major Depressive Disorder (MDD) is difficulty concentrating and increased distractibility. One form of distraction that occurs in everyday life is mind-wandering. The current study aims to test how individuals with MDD and healthy controls differ in their mind-wandering in everyday life.

**Methods:**

Adults diagnosed with MDD (*n* = 53) and healthy controls (n = 53) completed a week of experience sampling, with prompts administered up to eight times per day. At each prompt, participants reported the occurrence and characteristics of their mind-wandering. They also reported levels of momentary negative affect (NA), positive affect (PA), and rumination.

**Results:**

MDD participants reported mind-wandering almost twice as often as healthy control participants. Compared to healthy participants, MDD participants rated their mind-wandering as more negative, but did not differ in terms of temporal orientation. Higher NA and lower PA predicted mind-wandering in the MDD group but not healthy controls, even after controlling for rumination. Time-lagged analyses revealed that current mindwandering predicted future levels of PA in MDD participants but not in healthy controls; in contrast, current NA and PA did not predict future mind-wandering.

**Limitations:**

Limitations include our examination of specific forms of mind-wandering (i.e., we did not sample the full spectrum of this construct).

**Conclusions:**

Individuals with MDD frequently report engaging in mind-wandering in everyday life, and this appears to be coupled with affect. Mind-wandering may have maladaptive effects in MDD and could serve as a target for intervention.

## 1. Introduction

Major Depressive Disorder (MDD) is characterized by a range of cognitive difficulties. Specifically, individuals with MDD often report problems with executive functioning and memory (for reviews Lee et al., 2012; Pan et al., 2019). Several studies have also shown that individuals with MDD exhibit attentional difficulties: A central complaint, and a diagnostic criterion, in MDD is difficulty or diminished ability to think or concentrate (American Psychiatric Association, 2013). Attentional issues in the domains of selective attention, divided attention, and sustained attention have been reliably demonstrated in MDD (for a review see Keller et al., 2019). It is important to recognize, however, that attentional difficulties can also manifest from internal distractions in the form of mind-wandering.

Mind-wandering has been described as a shift of attention from one's external environment and their current goals towards their internal stream of consciousness (Smallwood and Schooler, 2006). It is important to note that mind-wandering, which can vary in its emotional and temporal content (see Welhaf et al., 2020), represents a form of “task-unrelated” thought that differs from a common symptom of MDD, rumination. Specifically, whereas rumination is characterized by deliberate, perseverative, negative thoughts (Nolen-Hoeksema, 2000; Treynor et al., 2003), mind-wandering is viewed as more spontaneous and flexible. Recently, researchers have proposed that mind-wandering and more perseverative forms of cognition, like rumination, are at opposite ends of a continuum of self-generated thoughts (Ottaviani et al., 2013).

Studies sampling daily life experiences suggest that young adults spend 30–50 % of their waking hours engaged in mind-wandering (e.g., Kane et al., 2007, 2017; Killingsworth and Gilbert, 2010). While mindwandering appears to be a relatively common experience, we know little about how mind-wandering manifests in MDD, especially in everyday life (Chaieb et al., 2022). The primary aim of the present study was to examine differences between healthy controls and individuals diagnosed with MDD in mind-wandering in daily life.

### 1.1. Mind-wandering and depressive symptoms

The links between mind-wandering and negative emotions and depressive symptoms are well established (for a review see Konjedi and Maleeh, 2017). In a seminal study, Giambra and Traynor (1978) found in a small sample of non-clinical individuals that there was a significant positive association between frequency of self-reported daydreaming (a form of mind-wandering) and depressive symptoms (see also Marchetti et al., 2014; Seli et al., 2019). Recent correlational research has extended this finding into the context of mind-wandering during an ongoing task. For example, in healthy individuals, higher levels of depressive symptoms have been associated with more frequent mind-wandering during a reading task (Watts and Sharrock, 1985) and a sustained attention task (Murphy et al., 2013; Nayda and Takarangi, 2021; Smallwood et al., 2007; Stawarczyk et al., 2013). The previously discussed research demonstrated that individual differences in depression are associated with frequency of mind wandering in healthy controls and provides supportive evidence to suggest that mind wandering might be more likely to occur in individuals with clinically diagnosed MDD.

### 1.2. Frequency and characteristics of mind-wandering in MDD

Evidence from laboratory studies demonstrates that individuals with MDD appear to mind wander more than healthy controls. Hoffmann et al. (2016) had individuals with MDD and healthy controls complete a choice reaction time task during which participants were asked periodically to report on the contents of their thoughts including temporal orientation, self- vs. other-orientation, and emotional valence (Engert et al., 2014; Ruby et al., 2013). The MDD participants in this study reported more mind-wandering during the task than did the healthy controls. Thus, individuals with MDD appear to have more difficulty than do healthy controls staying focused on the “here and now.”

These findings are consistent with an early theoretical view of mindwandering, the *Current Concerns Hypothesis* (Klinger et al., 1973). According to this theory, individuals have goals or “current concerns” active in consciousness until they are resolved or forgotten. Mindwandering occurs because these unresolved goals become more salient than current task goals, capturing individuals' attention. While some of these concerns might be positive and helpful for generating a solution for the unresolved goal (Klinger, 2009), they may often be negative, suggesting a maladaptive consequence of mind-wandering (Marchetti et al., 2016). Indeed, it is likely that individuals with MDD are more prone to mind-wandering because their attention is more easily drawn to such concerns (see Marchetti et al., 2012; Ruehlman, 1985).

Personal concerns and goals are likely to be more strongly cued in individuals' everyday life settings than they are in a laboratory context, perhaps because of the greater salience of everyday life. Only one small study to date, however, has examined mind-wandering in everyday life in individuals diagnosed with MDD. Ottaviani et al. (2015) recruited 18 participants with MDD and 18 healthy controls who completed a day of experience sampling during which they were asked to report on their mind-wandering every 30 min. Although the groups did not differ in overall mind-wandering, participants with MDD reported more perseverative cognition (e.g., rumination) but also less non-perseverative mind-wandering compared to the healthy control group. Thus, a goal of the current study is to further explore how frequently mind wandering occurs in participants with MDD (versus healthy controls) in everyday life using a larger sample of participants and across several days of experience sampling.

Recent work has shown that examining the specific contents of individuals' mind-wandering can provide a more nuanced view of what types of thoughts occupy consciousness (Welhaf et al., 2020). Indeed, the *Content-Regulation Hypothesis* (Smallwood and Andrews-Hanna, 2013) argues that it is the content (not simply the occurrence) of mind-wandering that is more responsible for its outcomes in everyday life. In this context, researchers have examined the association between different contents of mind-wandering and depressive symptoms. Researchers have also found that, in healthy controls, mind-wandering is biased towards the past (especially during periods of negative mood, e. g., Smallwood and O'Connor, 2011) and is typically negatively valenced in nature (e.g., Banks et al., 2016; Banks and Welhaf, 2022). However, it is unclear how these specific mind wandering characteristics might vary as a function of clinical status, especially in everyday life. Previous lab-based studies of mind wandering in MDD have found that participants with MDD report more negative (and less positive) thoughts and that their thoughts tend to be more future and past-oriented compared to controls (Hoffmann et al., 2016). This demonstrates that individuals with MDD appear to show a bias in their thought contents, suggesting that specific forms of mind-wandering might be prevalent in MDD than healthy controls. A second aim of the present study is to better understand how specific qualities of mind wandering differ between healthy controls and individuals with MDD in everyday life.

### 1.3. Temporal associations between affect and mind wandering

Finally, mind-wandering has been found to occur concurrently with negative affect (NA) in daily life in non-clinical samples (e.g., Kane et al., 2007, 2017; Song and Wang, 2012). The nature of the association between mind-wandering and affect, however, is still unclear. Whereas some researchers have argued that NA acts as a *precursor* to mindwandering (e.g., Poerio et al., 2013; Song and Wang, 2012), others have suggested that NA is a *consequence* of mind-wandering (Killingsworth and Gilbert, 2010; but see Welz et al., 2018, for a failed replication). Given these findings, it is possible that the temporal association between NA and mind-wandering is stronger for individuals with MDD because of their heightened susceptibility to NA and rumination (Nolen-Hoeksema, 2000; Nolen-Hoeksema and Morrow, 1993).

While investigators have also examined the association between mind-wandering and positive affect (PA), results of these studies suggest a weaker link between these constructs than is the case for NA. In healthy adults, daily-life mind-wandering that was rated as being highly interesting was associated with higher levels of PA (Franklin et al., 2013; Schooler et al., 2014), suggesting that mind-wandering can serve an adaptive process (McMillan et al., 2013). However, several studies have reported null associations between PA and mind-wandering, or that lower PA is associated with more mind-wandering (complementing the findings on NA; Welz et al., 2018). Thus, more work is needed to gain a better understanding of the link between mind-wandering and PA, especially in clinical groups.

## 2. Current study

The extant literature suggests a compelling link between MDD and mind-wandering, but further research is needed to clarify the nature of the occurrence, forms, and consequences of mind-wandering in MDD. Several studies have relied on self-report measures, which are likely influenced by recall biases that distort the actual frequency of mindwandering, so it is important to capture mind-wandering in real time. It is also important to examine mind-wandering in samples of individuals diagnosed with MDD to characterize mind-wandering in people with significant impairment. Finally, it is critical to examine mind-wandering outside of the laboratory, in everyday life, to provide evidence of ecological validity regarding differences in mind-wandering between healthy controls and MDD. In contrast to Ottaviani et al.'s (2015) findings, we hypothesized that rates of mind-wandering in daily life will be higher in MDD participants than in healthy controls.

We also had several hypotheses regarding between-subject phenomenological characteristics of mind-wandering. Regarding temporal orientation, we hypothesized that participants with MDD would exhibit a bias towards mind wandering about the past than healthy controls. Past oriented mind wandering might reflect a form of rumination whereby participants are “stuck” on thoughts or experiences that occurred recently during their day. Thus, individuals with MDD, who are more prone to rumination, might also experience a greater frequency of past-oriented mind wandering. Regarding emotional valence, we hypothesized that participants with MDD would exhibit more negatively valenced mind-wandering than will healthy controls. Individuals with MDD typically experience an increased frequency of repetitive negative thinking. As above, given that repetitive negative thinking falls under the umbrella of spontaneous cognition, it is possible that negatively valenced mind wandering, another form of spontaneous cognition, will occur more frequently in individuals with MDD.

We also examined within-person associations between affect and mind-wandering. We hypothesized that levels of momentary affect will be associated with increased mind-wandering. Specifically, more negative moods (i.e., higher NA, lower PA) will be associated with a greater likelihood of mind-wandering, which will be stronger in MDD than in healthy controls because of their tendency to experience high levels of NA in everyday life.

In the current study, we examined whether mind-wandering and affect are temporally linked. Specifically, we examined first if mindwandering at one prompt predicted affect ratings at the subsequent prompt. We hypothesized that mind-wandering will predict higher levels of subsequent NA and lower levels of PA, and that this will be stronger in MDD individuals than in the healthy controls given the higher frequency of rumination and repetitive negative thinking in MDD. We also examined whether affect ratings at the one prompt predicted mind-wandering at the subsequent prompt. We hypothesized that higher NA (and lower PA) at the current prompt will predict subsequent mind-wandering. Again, we hypothesized this pattern will be stronger in MDD participants than in healthy controls. Testing these questions will allow us to elucidate the temporal associations among mind-wandering, affect, and MDD.

## 3. Methods

### 3.1. Participants and procedure

One hundred and six participants were recruited as part of a larger study (see Mata et al., 2012; Thompson et al., 2012, 2013). Participants were required to be between the ages of 18 and 40 and be native English speakers. Individuals were eligible to participate if they either (a) experienced no current or past mental health disorders (healthy control group; *n* = 53); or (b) were currently diagnosed with MDD (depressed group; n = 53) as assessed by the *Structured Clinical Interview for DSM*–*IV Axis I Disorders* (SCID-I; First et al., 2001). Eligibility for the depressed group also included a Beck Depression Inventory–II (BDI–II; Beck et al., 1996) score of 14 or more and an absence of Bipolar I or II diagnoses, alcohol– drug dependence in past 6 months, and psychotic disorders. In addition, healthy control participants were only included if they scored *<*9 on the BDI-II. Participants in the MDD group were required to score of 14 or more on the BDI–II and an absence of alcohol or drug dependence in the past six months, Bipolar I or II diagnoses, and psychotic disorders. All participants were recruited from the Ann Arbor, Michigan and Stanford, California areas through posted advertisements at local agencies and business, and online. Sample demographics are reported in detail elsewhere (Mata et al., 2012; Thompson et al., 2012), so we summarize them here. Participants in the MDD group were slightly older (*M* = 28.2, *SD* = 6.4) than controls (*M* = 25.4, *SD* = 6.4). However, the groups did not differ in gender (% Female: MDD = 72 %, Control = 68 %) or race (% White: MDD = 74 %, Control = 62 %). The groups also did not differ in the number of completed prompts. Healthy controls completed, on average, 42.43 (*SD* = 7.78) prompts while individuals with MDD completed, on average, 44.04 (*SD* = 7.58) prompts, *t*(103.93) = −1.075, *p* = .285, Cohen's *d* [95 % CI] = −0.21 [−0.59, 0.17]. Consistent with previous analyses of these data, participants were recruited equally at both sites but were pooled together for increased reliability and generalizability.

General experience sampling procedures on this sample have been described elsewhere (Mata et al., 2012; Thompson et al., 2012, 2013). In brief, participants were provided with a hand-held electronic device (Palm Pilot Z22) with Experience Sampling Program 4.0 (Barrett and Feldman Barrett, 2000) installed. Over the course of 7–8 days, participants were randomly prompted (via a tone on the device) eight times a day between 10:00 a.m. and 10:00 p.m. Thus, participants completed a max of 56 prompts over the course of the week. At an initial session, participants were trained on using the device and provided informed consent. Participants were compensated upon completion with a bonus for completing >90 % of the prompts. Institutional Review Boards and both the University of Michigan and Stanford University approved this protocol.

### 3.2. Measures

Our previous studies have examined various aspects of the experience sampling protocol. Below, we describe the measures within the protocol that are central to the main question regarding mindwandering, PA, and NA.

#### 3.2.1. Mind-wandering

The first item of each mind-wandering prompt asked participants whether they were mind-wandering at the time of the signal (“*At the time of the beep, my mind had wandered to something other than what I was doing*”). Participants answered with a *yes* or *no* response. If participants reported that they were mind-wandering, they then answered questions about their mind-wandering experience. For the current study, we specifically focus on two characteristics of mind wandering, emotional valence and temporal orientation. Specifically, the emotional valence question asked, “*I was thinking about something…*” and participants could choose from the following response options *positive, neutral*, and *negative*. For the temporal orientation question, participants were asked “*I was thinking about something in the…*” with possible response options of *the past, the present or the future*.

#### 3.2.2. Affect ratings

At each prompt, participants answered eleven items regarding their current PA (i.e., *I feel happy/excited/alert/active right now*) and NA (*I feel sad/anxious/angry/frustrated/ashamed/disgusted/guilty right now*). These words were derived from measures of mood and affect including the Positive and Negative Affect Schedule (Watson et al., 1988) and Ekman's basic emotions (Ekman et al., 1972). All items were answered on a 4-point scale ranging from 1 (*not at all*) to 4 (*a great deal*). For each survey we calculated a PA and NA composite score by averaging the item scores. We calculated between- and within-person reliability ω using the *multilevelTools* package (Wiley, 2024). For PA, between-person reliability was 0.920 and within-person reliability was 0.747. For NA, between-person reliability was 0.969 and within-person reliability was 0.829. These within-person reliability estimates were in the range of moderate to substantial (Shrout, 1998). Table 1 presents the intra-class correlation (ICC) for both composites.

#### 3.2.3. Rumination

At each prompt, participants completed a shortened version of the Ruminative Response Scale (Treynor et al., 2003), specifically, they reported on the five-item brooding scale (e.g., *I was thinking* “*What am I doing to deserve this?*”). All items were answered on a 4-point scale ranging from 1 (*not at all*) to 4 (*a great deal*). For each survey, we calculated a brooding score by summing the individual items. Between-person reliability was 0.973 and within-person reliability was 0.804 indicating moderate within-person reliability. Table 1 presents the ICC for the brooding score. Rumination items were answered regardless of participants' response to the mind wandering question.

### 3.3. Analytic plan

Our first analysis focused specifically on comparing overall rates of mind-wandering in daily life between control and MDD groups. To test this, we calculated, for each participant, their rate of mind-wandering over the week. We then used a *t*-test to compare the two groups. We also examined differences in mind-wandering at a within-person level. Because our mind-wandering outcome was binary (i.e., mind-wandering was either reported or not), we used hierarchical generalized linear modeling (HGLM) with the binomial distribution as the sampling model at Level 1 and the logit function to transform predicted values. Thus, our predictors are reported on the logit scale, meaning that they represent the natural log of the odds of reporting mind-wandering. We exponentiated the logit values to transform them into probabilities across a range of values for predictors that were significantly associated with the likelihood of mind-wandering. These HGLM models were conducted in R (R Core Team, 2023) using the *lmer* package (Kuznetsova et al., 2017). Group was included as a Level 2 (between-person) predictor and was dummy coded (healthy control group = 0; MDD group = 1).

Our second analysis focused on the between-subject phenomenological characteristics of mind-wandering. For the “surprised” question, we calculated the average rating of surprise at each mind-wandering report, for each participant. These were again compared using a t-test. For the emotional valence and temporal orientation reports, we examined differences in the frequency of each type of mind-wandering. These temporal orientation and emotional valence data were analyzed using a 2 (Group, a between subject factor) × 3 (Mind-wandering Category, a within subject factor) mixed model ANOVA. For these analyses, we focused only on surveys when participants reported mind-wandering and thus, the denominator varied for each participant. There were five participants in the healthy control group and one participant in the MDD group who never reported mind-wandering, as such they were excluded from these analyses.

We next examined contextual or in-the-moment predictors of mindwandering. For these analyses, we again used multilevel modeling given the nested nature of the data (i.e., prompts were nested within participants). All multilevel models were conducted using the *lme4* package (Bates et al., 2015). *P*-values for these multi-level models were calculated using the *lmerTest* package (Kuznetsova et al., 2017). To provide readers with a plausible range of population effect sizes, we provide 95 % confidence intervals of all effect sizes of interest, as recommended by Hoenig and Heisey (2001), Levine and Ensom (2001) and O'Keefe (2007).

Level 1 predictors included the NA or PA composites at each prompt. Level 1 predictors were person-centered and can be interpreted as instances with higher (vs. lower) NA or PA, relative to the individuals' average rating. Group was included as a Level 2 (between-person) predictor and was dummy coded (Control = 0; MDD = 1). All models included random intercepts and slopes.

Finally, in line with previous work (e.g., Poerio et al., 2013; Welz et al., 2018), we also examined time-lagged effects of affect and mind-wandering. For all models, we limited analyses to within-day associations (i.e., time *t* responses did not predict time *t* + 1 if the prompt occurred on the next day). First, we tested if affect ratings (negative or positive) at time *t* predicted mind-wandering at time *t* + *1*. In a separate analysis, we examined if being on-task or mind-wandering at time *t* predicted future affect levels (negative or positive in separate models) at time *t* + *1*. We included time *t* affect/mind-wandering as fixed effects in the respective models to account for the autocorrelation between time *t* and time *t*_+1_ variables (Ruby et al., 2013; Welz et al., 2018).

## 4. Results

### 4.1. Do rates of mind-wandering in daily life vary across control and MDD participants?

Table 1 provides the descriptive statistics for all the variables of interest for the current study for both the healthy control and MDD groups. Table 2 provides the within- and between-person correlations among the variables (both overall and by condition).

**Fig. 1.**
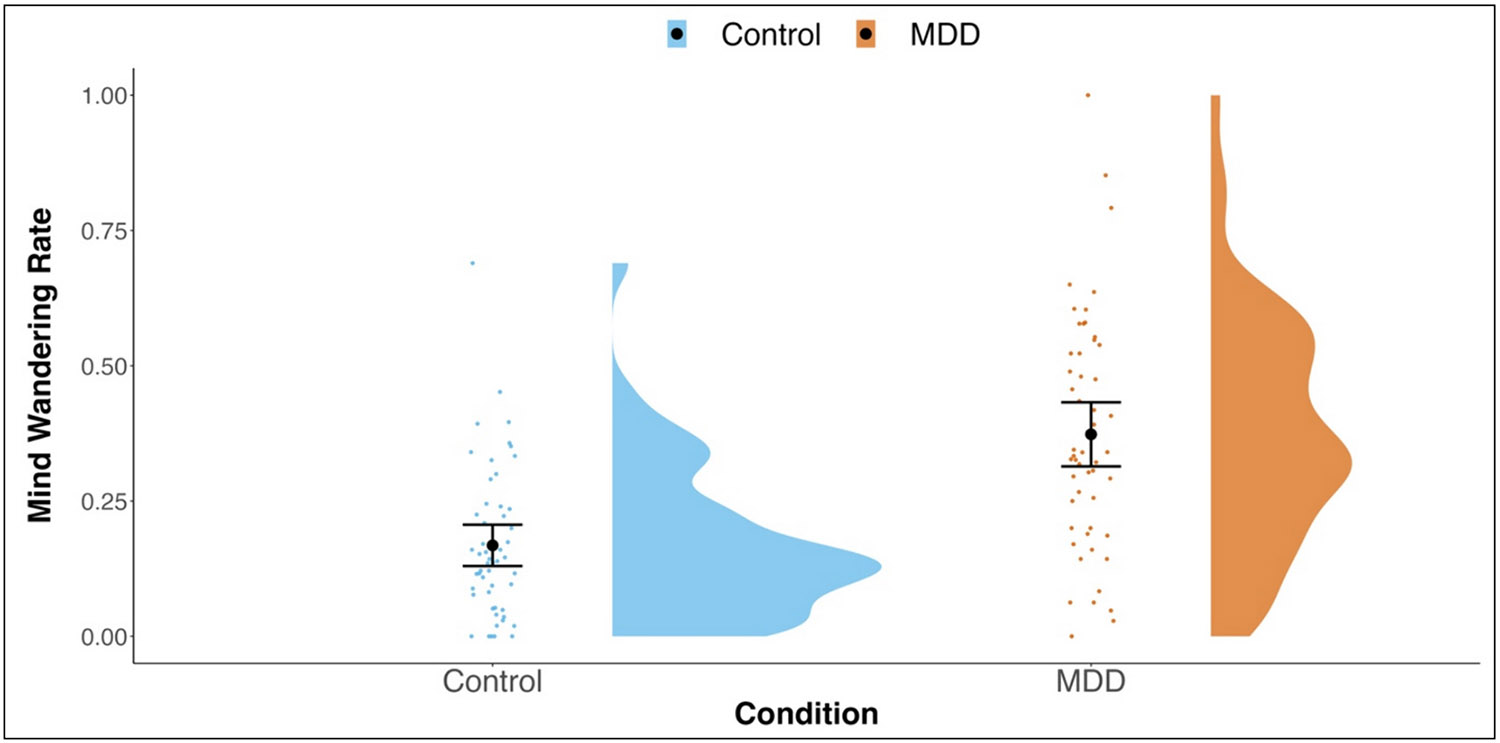
Raincloud plots (Allen et al., 2021) depicting differences in Daily Life Mind-wandering by Group. Individual dots represent participant values with the corresponding distribution. Closed circles reflect the group means and the corresponding 95 % confidence interval.

Our first question focused on examining whether mind-wandering in daily life differs based on participants' MDD status. As seen in Table 1, over the week of experience-sampling, the healthy control group reported mind-wandering on 17 % of the prompts. In contrast, the MDD group reported mind-wandering over twice as often, at 37 %, *t*(88.82) = −5.845, *p* < .001, Cohen's *d* [95 % CI] = −1.14 [−1.54, −0.72] (see Fig. 1).

To examine the proportion of variance in daily-life mind-wandering that was attributable to within- and between-person levels, we conducted an intercept-only model (Raudenbush and Bryk, 2002), with prompt-level mind-wandering as the dependent variable and no Level 1 or Level 2 predictors. The intraclass correlation of the intercept-only model was 0.322, indicating that 68 % of the variance in daily-life mind-wandering was at the within-person level and 32 % was attributable to between-person differences. We next tested if group status significantly predicted momentary mind-wandering. Consistent with the group level analyses, individuals with MDD were more likely to report mind-wandering at the individual prompt, OR = 0.30 [95 % CI = 0.19, 0.46], *b* = −1.22, SE = 0.22, *z* = −5.474, *p* < .001. Inspection of Fig. 1 shows not only that MDD reported higher levels of mind-wandering in daily life, but also that the overall distribution of mind-wandering rates for the MDD participants was larger than that for the controls, Levene's Test *F*(1,104) = 8.418, *p* = .005.

### 4.2. Phenomenological differences in mind-wandering between control and MDD participants

We next examined overall differences in the temporal orientation of participants' mind-wandering. There was no effect of group, *F*(1,98) = 0.17, *p* = .683, partial h^2^ = 0.002 [90 % CI = 0.000, 0.038], but there was a significant effect of temporal orientation, *F*(1.64,160.25) = 69.55, *p* < .001, partial h^2^ = 0.415, [90 % CI = 0.318, 0.492]. There was no significant group × temporal orientation interaction, *F*(1.64,160.25) = 1.68, *p* = .194, partial h^2^ = 0.017, [90 % CI = 0.000, 0.058]. Irrespective of group, when participants reported mind-wandering, it was most often focused on the present (*M* = 58 %, *SD* = 26 %), followed by the future (*M* = 30 %, *SD* = 24 %), and then the past (*M* = 12 %, *SD* = 7 %).

**Fig. 2.**
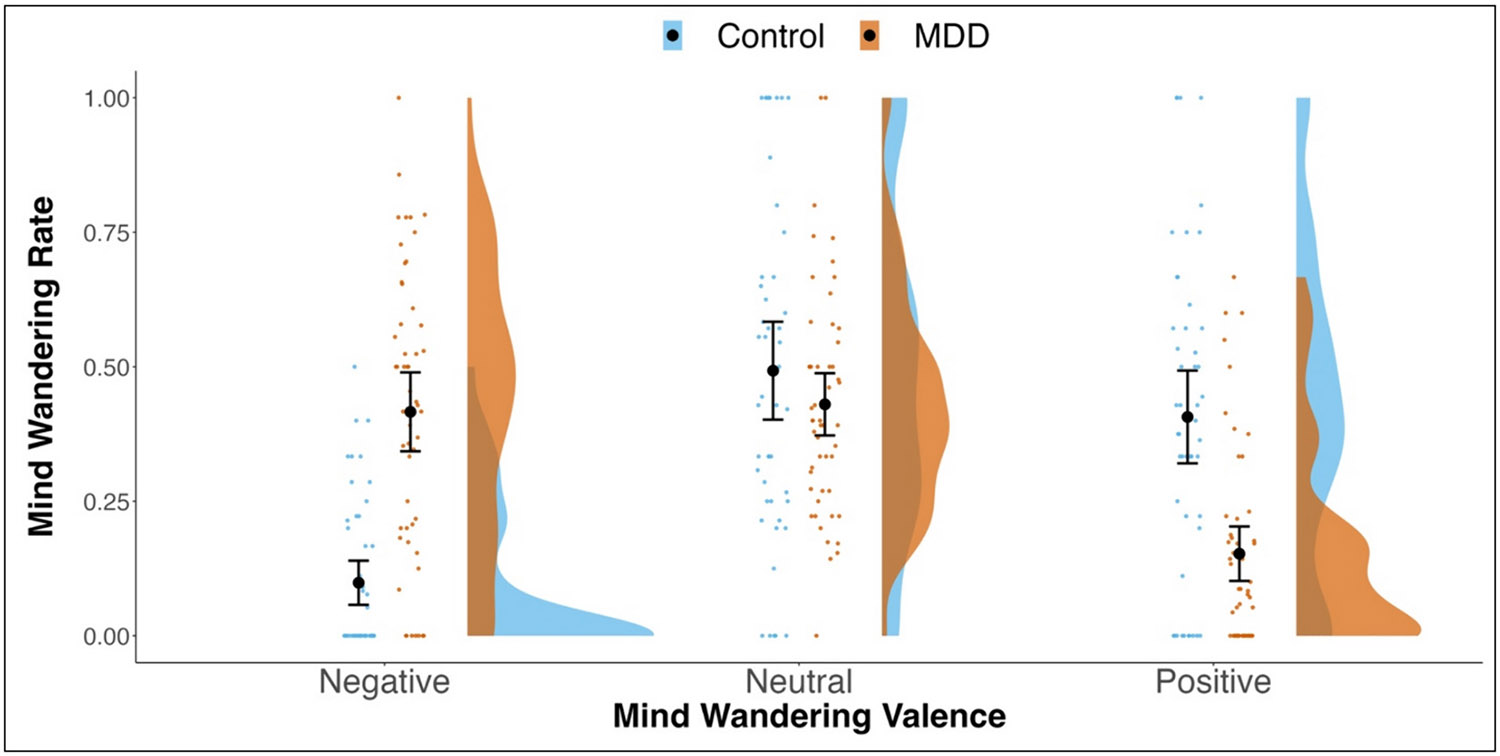
Raincloud plots (Allen et al., 2021) depicting differences in Emotional Valence of Daily Life Mind-wandering by Group. Individual dots represent participant values with the corresponding distribution. Closed circles reflect the group means and the corresponding 95 % confidence interval.

Finally, we examined group differences in the emotional valence of participants' mind-wandering. Again, there was no significant effect of group, *F*(1,98) = 0.17, *p* = .683, partial h^2^ = 0.002 [90 % CI = 0.000, 0.038]. There was a significant effect of emotional valence, *F* (1.89,185.38) = 14.34, *p* < .001, partial h^2^ = 0.127 [90 % CI = 0.057, 0.198], which was qualified by a significant interaction of group and emotional valence, *F*(1.89,185.38) = 24.19, *p* < .001, partial h^2^ = 0.198 [90 % CI = 0.114, 0.274] (see Fig. 2).

Follow-up tests showed that the healthy control and MDD groups did not differ in their frequency of neutrally valenced mind-wandering, *t* (98) = 1.183, *p* = .240, Cohen's *d* [95 % CI] = 0.24 [−0.16, 0.64]; however, there were significant differences in the rate of both negative, *t* (98) = −7.426, *p* < .001, Cohen's *d* [95 % CI] = −1.50 [−1.95, −1.05], and positive mind-wandering, *t*(98) = 5.204, *p* < .001, Cohen's *d* [95 % CI] = 1.05 [0.63, 1.47]. The MDD group reported negatively valenced mind-wandering on 42 % (*SD* = 26 %) of their mind-wandering reports, while healthy control group reported negatively valenced mindwandering on only 10 % (*SD* = 14 %) of their mind-wandering episodes. Similarly, the MDD group reported on average, positively valenced mind-wandering episodes on 15 % (*SD* = 18 %) of the prompts compared to the healthy control group's average of 41 % (*SD* = 30 %).

### 4.3. Current PA and NA as predictors of mind-wandering in daily life

**Fig. 3.**
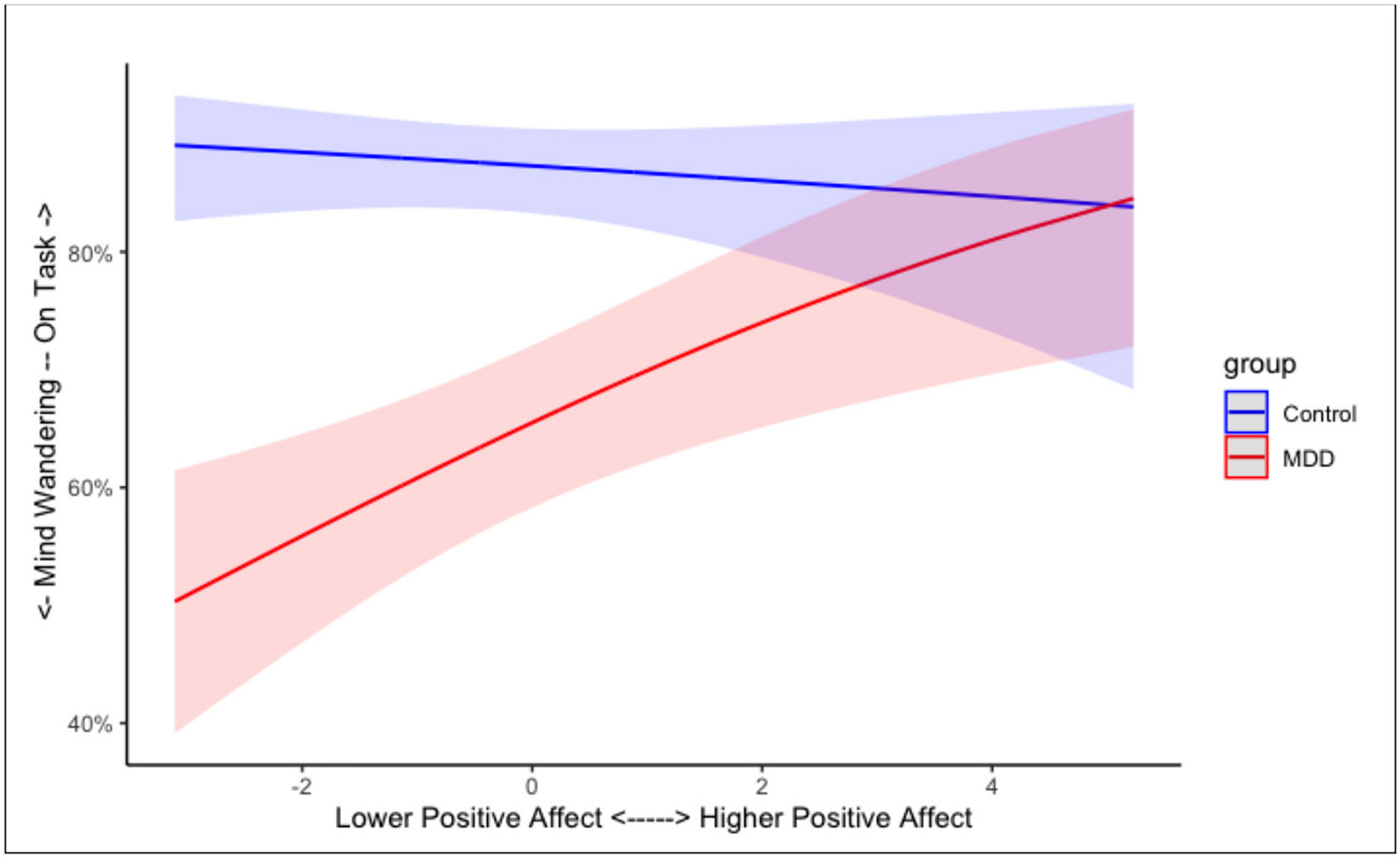
Predicted effect of momentary PA on mind-wandering for both healthy control and MDD groups. Shaded regions represent the 95 % confidence interval for each group.

Contemporaneous ratings of PA did not predict the occurrence of mind-wandering, OR = 0.95 [95 % CI = 0.82, 1.10], *b* = −0.05, SE = 0.08, *z* = −0.708, *p* = .479. There was, however, a significant interaction between group and PA ratings, OR = 1.29 [95 % CI = 1.07, 1.56], *b* = 0.26, SE = 0.10, *z* = 2.657, *p* = .008. As shown in Fig. 3, when participants (regardless of group) reported higher levels of PA at a prompt, they were more likely to report being on-task; however, at lower levels of PA, the MDD group was more likely to report mind-wandering than was the healthy control group.

**Fig. 4.**
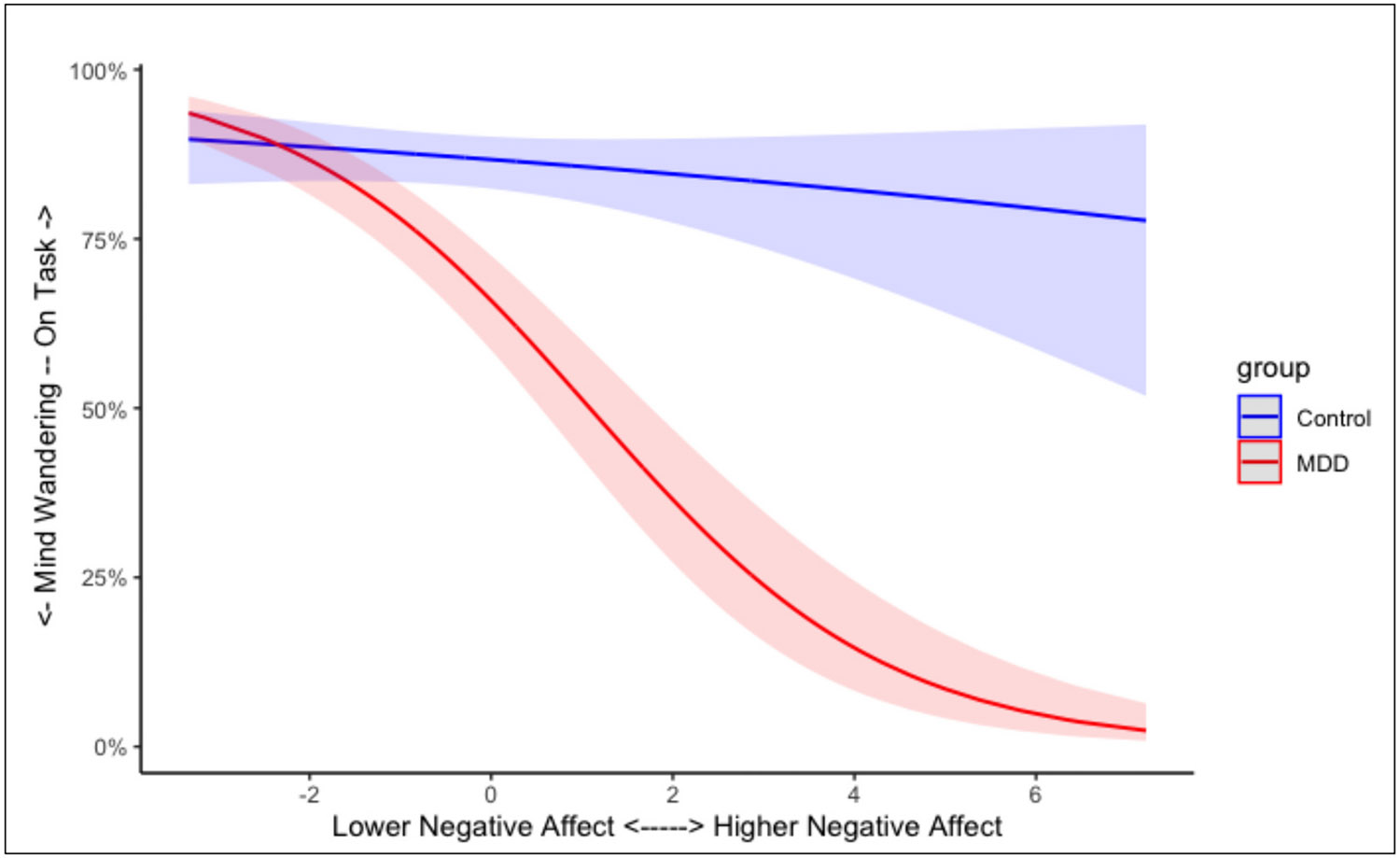
Predicted effect of momentary Negative Affect on mind-wandering for both healthy control and MDD groups. Shaded regions represent the 95 % confidence interval for each group.

Contemporaneous NA was not associated with the occurrence of mind-wandering, OR = 0.92 [95 % CI = 0.79, 1.07], *b* = −0.09, SE = 0.08, *z* = −1.112, *p* = .262. Again, there was a significant interaction between group and NA, OR = 0.59 [95 % CI = 0.49, 0.73], *b* = −0.52, SE = 0.10, *z* = −5.076, *p* < .001. As shown in Fig. 4, when participants (regardless of group) reported lower levels of NA, they were more likely to report being on-task; however, at higher levels of NA, both groups, but especially the MDD group, were more likely to be mind-wandering.

### 4.4. Rumination as a predictor of daily life mind-wandering

Contemporaneous brooding levels did not predict the occurrence of mind-wandering, OR = 0.95 [95 % CI = 0.82, 1.10], *b* = −0.07, SE = 0.08, *z* = −0.824, *p* = .410. However, there was a significant interaction between group and brooding levels, OR = 0.57 [95 % CI = 0.47, 0.70], *b* = −0.56, SE = 0.11, *z* = −5.314, *p* < .001. As with the affect models, when participants (regardless of group) reported lower levels of brooding at a prompt, they were more likely to report being on-task. However, at higher momentary levels of brooding, the MDD group was more likely to report mind-wandering compared to the healthy control group.

We also conducted the affect models including contemporaneous brooding as a covariate. That is, we tested if the interaction between affect and group status still predicted mind-wandering after controlling for levels of brooding. In both cases the group × affect interaction was still significant: NA OR = 0.61 [95 % CI = 0.49, 0.74], *b* = −0.50, SE = 0.10, *z* = −4.770, *p* < .001; PA: OR = 1.24 [95 % CI = 1.02, 1.50], *b* = 0.21, SE = 0.10, *z* = 2.181, *p* = .029.

### 4.5. Time-lagged analyses

#### 4.5.1. Does current affect predict future mind-wandering?

In a model with PA at time *t* predicting mind-wandering at time *t* + 1, only the main effect of group was significant, OR = 3.34 [95 % CI = 2.14, 5.20], *b* = 1.20, SE = 0.23, *z* = 5.321, *p* < .001. Neither mindwandering at time *t*, OR = 1.02 [95 % CI = 0.82, 1.27], *b* = 0.02, SE = 0.11, *z* = 0.219, *p* = .827, nor PA at time *t*, OR = 1.09 [95 % CI = 0.95, 1.27], *b* = 0.09, SE = 0.08, *z* = 1.235, *p* = .217, predicted mindwandering at time *t* + 1. Furthermore, the interaction between group and PA at time *t* was not significant, OR = 0.90 [95 % CI = 0.74, 1.08], *b* = −0.11, SE = 0.10, *z* = −1.147, *p* = .251.

In a model with NA at time *t* predicting mind-wandering at time *t* + 1, the main effect of group was significant, OR = 3.16 [95 % CI = 2.03, 4.92], *b* = 1.15, SE = 0.23, *z* = 5.112, *p* < .001. NA at time *t* did not predict future mind-wandering at *t* + 1, OR = 1.04 [95 % CI = 0.89, 1.19], *b* = 0.03, SE = 0.07, *z* = 0.415, *p* = .678. Mind-wandering at time *t* did not predict mind-wandering at *t* + *1*, OR = 0.95 [95 % CI = 0.76, 1.19], *b* = −0.05, SE = 0.12, *z* = −0.453, *p* = .650. Finally, the interaction between group and NA at time *t* was not significant, OR = 1.15 [95 % CI = 0.95, 1.38], *b* = 0.14, SE = 0.09, *z* = 1.438, *p* = .150.

#### 4.5.2. Does current mind-wandering predict future affect?

PA at time *t* + *1* was significantly predicted by PA at time *t*, *b* = 0.26, SE = 0.03, *t*(3027.00) = 7.960, *p* < .001. Mind-wandering at time *t* did not predict PA at time *t* + *1*, *b* = −0.01, SE = 0.07, *t*(3027.00) = −0.282, *p* = .778. Group status did predict PA at time *t* + *1*, *b* = −0.08, SE = 0.04, *t*(3027.00) = −2.020, *p* = .043. The interaction between current mindwandering and group status predicted PA at *t* + *1*, *b* = 0.17, SE = 0.08, *t* (3027.00) = 2.079, *p* = .038. Individuals with MDD who reported mindwandering at the current prompt had reduced PA at the next prompt; in contrast, healthy controls had similar levels of PA at the future prompt regardless of their current mind-wandering.

NA levels at time *t* significantly predicted NA time *t* + *1*, *b* = 0.20, SE = 0.02, *t*(2959.00) = 11.175, *p* < .001. Neither participants' mindwandering at time *t*, *b* = 0.07, SE = 0.07, *t*(2959.00) = 0.98, *p* = .329, nor group status predicted NA at time *t* + *1*, *b* = 0.05, SE = 0.04, *t* (2959.00) = 1.200, *p* = .230. Finally, the interaction between current mind-wandering and group status did not predict NA at time *t* + 1, *b* = −0.14, SE = 0.08, *t*(2959.00) = −1.678, *p* = .094.

## 5. Discussion

Difficulty concentrating can underlie other cognitive problems associated with MDD, including impaired learning and memory, processing speed, and executive function (Pan et al., 2019). We used experience sampling to test whether individuals diagnosed with MDD differed from healthy controls in the frequency and characteristics of their mind-wandering in everyday life. Our results suggest several unique associations between MDD and mind-wandering. Consistent with our hypotheses, individuals with MDD reported nearly twice as high a frequency of mind-wandering as did healthy controls, supporting the result of previous laboratory studies that have documented differences in mind-wandering between healthy controls and MDD groups (Hoffmann et al., 2016). However, the results of the current study counter those reported by Ottaviani et al. (2015), who found no differences between MDD and healthy controls in their overall experience of mind-wandering in everyday life. One possible explanation for this difference involves the period during which participants evaluated their ongoing thoughts. In our study, participants reported whether they felt their mind was wandering immediately before they received the prompt. Ottaviani et al. also asked participants to report what they were just thinking about prior to the beep, but they also allowed participants to report how long they were engaged in such thoughts (e.g., prior 5, 10, 20 min or since the last beep). People may not be particularly cognizant of their mind-wandering and asking them to think back over several minutes may render their reports less reliable (e.g., Kane et al., 2021).

Examining the content of participants' thoughts revealed several unique patterns. Regarding temporal orientation, we did not find any evidence that future- or past-oriented mind-wandering was more prevalent in MDD, which contrasts previous lab studies of mind wandering qualities in individuals with MDD (e.g., Hoffmann et al., 2016). While negative mood has often been associated with a bias towards mindwandering about past-related thoughts (at least in healthy adults, Poerio et al., 2013; Stawarczyk et al., 2013), we did not find evidence for such a past-oriented bias. Thus, participants with MDD did not appear to be “stuck” on previous thoughts unrelated to what they were currently engaged in. Past-oriented mind wandering then, might be a different feature of spontaneous thought than rumination that is commonly seen in MDD. However, the frequency of past-oriented mind wandering was relatively low in the sample, and so, future work will need to further examine the link between temporal orientation of mind wandering in MDD in daily life to more fully understand this association.

In terms of the valence of internal thoughts, we examined if individuals with MDD showed a bias towards negatively valenced mindwandering, consistent with views of thought patterns in MDD in previous work (e.g., Hoffmann et al., 2016; Marchetti et al., 2012; Nolen-Hoeksema and Morrow, 1993). Consistent with our hypothesis, the MDD group reported mind-wandering that was more negatively valenced than the healthy control group. Repetitive negative thinking is commonly demonstrated in MDD (Mineka et al., 1998), and similar patterns of negatively valenced thoughts seem to occur during mind-wandering in everyday life. It is worth noting that there was also considerable individual differences in the frequency of negatively valenced mind wandering, especially in MDD (indicated by the larger spread of the distribution). That is, some individuals with MDD exhibit relatively few instances of negative mind wandering, while others reported that almost all their mind wandering was negative. Future work should consider what other factors might explain variation in negatively valenced mind wandering within participants with MDD. The results of the emotional valence analysis do suggest, though, that while mind-wandering is more frequent in MDD, this might be specific to negatively valenced mindwandering.

The occurrence of mind-wandering has been shown to be influenced by previous mood and to predict future mood (e.g., Killingsworth and Gilbert, 2010; Poerio et al., 2013). That is, mind-wandering is associated with subsequent increases in NA, and increased levels of NA lead to increased likelihood of mind-wandering in future. However, our hypothesis that this possible bidirectional association between mindwandering and NA would be evident, and perhaps stronger, in individuals with MDD was only partially supported. In contrast to our hypotheses, we did not find any evidence for a bidirectional temporal association between mind-wandering and NA. Surprisingly, we did find that current mind-wandering was associated with future PA for MDD participants. Thus, the temporal effects of mind-wandering in MDD might be specific to reduced PA, further highlighting the importance of considering PA (and its predictors) in this disorder (Garland et al., 2010).

### 5.1. Clinical implications

Our findings have clinical relevance. For example, several therapeutic approaches in MDD, including Cognitive Behavioral Therapy (Spinhoven et al., 2018) and Mindfulness Meditation (Feruglio et al., 2021), focus on being aware of and changing patterns of thinking that might be maladaptive. Our results suggest that such approaches could be used to pinpoint experiences of mind-wandering in MDD, especially those that are negative in content. Our results also suggest that individuals with MDD are no less aware of their thoughts than are healthy controls, so therapies could be tailored to make those with MDD more aware of the *content* of their thoughts rather than just their occurrence, especially in their everyday lives.

### 5.2. Limitations and constraints on generalizability

We should note two limitations of the present study. First, we did not sample other aspects of mind-wandering and task-unrelated thoughts such as intentionality (Seli et al., 2016, 2019) or how freely-moving participants' thoughts were in the moment (Mills et al., 2018, 2021). In healthy controls, both dimensions of spontaneous cognition appear to be associated with symptoms of depression and affect. Assessing these different dimensions of off-task thought could provide a more nuanced view of how mind-wandering occurs in MDD. For example, we would expect that individuals with MDD might be more prone to mindwandering that occurs unintentionally (Seli et al., 2019) or more less freely-moving (i.e., more constrained) mind-wandering (Christoff et al., 2016; Smith et al., 2022). Second, the sample was composed of adults between the ages of 18 and 40. Older adults frequently report less mindwandering than do their younger peers (Jordão et al., 2019), so it will be important to replicate our findings in a sample of older adults. Third, the measures of NA and PA used in this study did not fully represent the affective circumplex as assessed in other EMA studies (e.g., Pietromonaco and Barrett, 2009). Consequently, we were unable to examine how associations between mind wandering and NA and PA may differ based on arousal. Finally, although our hypotheses were generated and grounded in theoretical and empirical findings in the mind-wandering and depression literatures, this study was not pre-registered; therefore, future research should be conducted to replicate these findings, with a pre-registered analytic plan.

## 6. Conclusions

Despite these limitations, the present study provides insight about the association between MDD and mind-wandering, a naturally occurring phenomenon that accounts for 30–50 % of our waking lives. Individuals with MDD reported mind-wandering nearly twice as often as healthy controls, and this difference seemed to be driven by higher levels of negatively valenced mind-wandering. However, healthy controls and MDD participants did not differ in the temporal orientation or awareness of their mind-wandering. Concurrent levels of affect (lower PA and higher NA) predicted the likelihood of mind-wandering in individuals diagnosed with MDD but not in healthy controls, suggesting that current affective experiences are coupled with distracting thoughts in depression. Increased NA also predicted future mind-wandering, suggesting a cognitive link between individuals' momentary emotional states and their likelihood of being distracted at a future time; this association may be an important target for intervention.

## Figures and Tables

**Table 1:** Descriptive Statistics for variables in the current study.

Variable	Control Group (*N* = 53)			MDD Group (N = 53)			ICC
	Mean	Within SD	Between SD	Mean	Within SD	Between SD	
Mind-wandering	0.17	0.34	0.14	0.37	0.44	0.21	0.32
NA	1.14	0.21	0.17	1.88	0.54	0.52	0.62
PA	2.15	0.52	0.47	1.68	0.51	0.39	0.46
RSS Brooding	5.31	0.79	0.47	8.23	2.78	2.79	0.58

*Note*. Items for NA, PA, and RSS were rated on a 1–4 scale. NA = Negative Affect Composite; PA = Positive Affect Composite; RSS = Ruminative Response Scale.

**Table 2:** Correlations (between- and within-participant) for the current study in the overall sample and within each group.

		Mind- wandering	NA	PA	RSS Brooding
Overall Sample	Mind-wandering		0.45***	−0.19^^^	0.47
	NA	0.20***		−0.32***	0.79
	PA	−0.03^^^	−0.09***		−0.11
	RSS Brooding	0.21	0.63	−0.07	
Control	Mind-wandering		0.21	0.09	0.27^^^
	NA	0.03		0.13	0.83***
	PA	0.03	−0.08***		0.13
	RSS Brooding	0.06**	0.49***	−0.02	
MDD Group	Mind-wandering		0.12	0.10	0.24^^^
	NA	0.27***		0.01	0.66***
	PA	−0.07**	−0.11***		0.38**
	RSS Brooding	0.26***	0.65***	−0.09***	

*Notes*. Between-Subject correlations presented above diagonal. Within-Subject Correlations presented below diagonal. NA = Negative Affect Composite; PA = Positive Affect Composite; RSS = Ruminative Response Scale.

^ *p* < .10.

** *p* < .01.

*** *p* < .001.
